# Red Color and Risk-Taking Behavior in Online Environments

**DOI:** 10.1371/journal.pone.0134033

**Published:** 2015-07-24

**Authors:** Timo Gnambs, Markus Appel, Aileen Oeberst

**Affiliations:** 1 Institute of Psychology, Osnabrück University, Osnabrück, Germany; 2 Psychology Department, University of Koblenz-Landau, Landau, Germany; 3 Leibniz-Institut für Wissensmedien, Tübingen, Germany; Tsinghua University, CHINA

## Abstract

In many situations red is associated with hazard and danger. As a consequence, it was expected that task-irrelevant color cues in online environments would affect risk-taking behaviors. This assumption was tested in two web-based experiments. The first study (*N* = 383) demonstrated that in risky choice dilemmas respondents preferred the less risky option when the displayed university logo was in red (versus gray); but only when both choice alternatives were at least moderately risky. The second study (*N* = 144) replicated these results with a behavioral outcome: Respondents showed more cautious behavior in a web-based game when the focal stimuli were colored red (versus blue). Together, these findings demonstrate that variations in the color design of a computerized environment affect risk taking: Red color leads to more conservative choices and behaviors.

## Introduction

People constantly have to make decisions in their daily lives, ranging from, for example, what to have for lunch or whether to put one’s money into a bank account or invest it in a risky stock portfolio. With the advent of the Internet, an increasing number of decisions are also made online. This includes whether or not to disclose sensitive information about oneself in an online social network or to provide credit card details in an online shop. People’s choices are typically not exclusively determined by careful cost-benefits considerations of the available choice alternatives [1, 2]. Rather, the characteristics of an online or offline environment and individual differences contribute to the choices they make (see [3] for a review). A prominent situational feature which is ubiquitous in our surroundings is color. Colors not only shape individuals’ aesthetic feelings; they also have pronounced psychological implications. For example, research has shown that subtle red color cues can inhibit cognitive performance [4], increase dominance in competitive interactions [5], and modulate people’s mating behavior [6]. In many situations red is associated with danger and perceptions of threat [7, 8]. Despite the well-established link between red and danger, there is still a lack of experimental studies examining the effects of color on risk-taking behavior. Therefore, the present study contributes to the growing body of research on color psychology by examining the behavioral effects of red on risk taking in computerized environments. Two web-based experiments demonstrate that minor aesthetic variations in the color design of a website affect people’s decisions between risky choice alternatives (Study I) and risk-taking behavior in a web-based game (Study II).

### Psychological Effects of Color in Online Environments

Increasing evidence suggests that colors carry meaning and, depending on the prevalent situational conditions, differentially affect behaviors and psychological functioning (see [9] and [10] for reviews). For example, in achievement situations (e.g., during test taking) red triggers avoidance motivation and, as a consequence, impairs test performance [11], whereas in potential mating situations red has the opposite effect and activates approach motivation by increasing sexual attractiveness [12]. A limitation of most studies on color psychology so far has been their predominant reliance on highly controlled lab settings. Although these studies managed to provide compelling evidence for a causal link between color and various psychological outcomes, they were unable to determine the relevance of these effects in more realistic situations with less controlled conditions. Only rather recently has applied research on the generalizability of the effect of the color red started to gain momentum. Field studies conducted in real world settings outside the laboratory demonstrated that women sitting at a bar were more frequently approached by men when they used red lipstick [6], patrons gave more tips to waitresses wearing red dresses [13] or lipstick [14], consumers ate less snack food from a red plate [15], and drivers experienced more aggression in traffic jams when seeing red cars [16]. All these studies concordantly demonstrated that inconspicuous color cues also have a pronounced influence on people’s behavior in a variety of applied settings with less experimental control.

However, one important area of everyday life has received less attention so far. A growing part of individuals’ working and private lives is shaped by computer technologies; for example, people increasingly connect with their friends in online social networks, they conduct business transactions via online banking, and they buy goods and services over the Internet [17, 18, 19]. In light of recent findings showing that people sometimes behave differently online than in real life settings [20, 21], it seems crucial to extend applied research on color effects to online environments. A few studies indicate that red shows similar effects on the Internet. For example, unobtrusive design elements in a web-based questionnaire (e.g., color variations in the progress bar) affected male respondents’ performance in a knowledge test [4]. Similarly, in virtual multiplayer games seeing red is a powerful psychological distractor and decreases the probability of competitive success [22]. Moreover, in online social networks red increased contact requests to personal ads [23] and propagated the diffusion of user-generated content [24]. Finally, in the financial realm red influenced the bids in online auctions [25] and click rates on web-based banner ads [26]. Taken together, a small but growing number of studies suggests that subtle variations in the design of the online environment can also matter outside the laboratory when environmental differences are less controlled.

### Red Color and Risk-Taking

Despite the convincing evidence that colors also have pronounced behavioral effects in online environments [4, 22, 25, 26], their impact on risk-taking behavior has not yet been explicitly investigated. The lack of empirical research in this area is somewhat surprising since in many situations red is associated with hazard and danger as reflected, for example, in the color of human blood or glowing embers. Consequently, red is typically used as a universal signal of warning such as in traffic lights. Experimental studies even suggest an implicit association between red color and danger; that is, subtle presentations of red color cues automatically activate danger-related cognitions [27]. As a consequence, in competitive interactions seeing red enhances the perception of threat and thus tends to impair one’s performance [5, 8, 28]. Due to the association of the color red with threat, red color was found to affect the processing of persuasive health messages [29]; for example, red increased people’s willingness to be vaccinated [30]. So far, there have been few studies examining the effects of color on decision making between risky outcomes.

Most research on the behavioral consequences of red color is based on the assumption that red triggers avoidance motivation and thus impairs performance in achievement situations [31] or increases the salience of financial losses as compared to financial gains [28]. Due to its implicit association with danger [27], red color is likely to shift individuals’ attention involuntarily to potential losses associated with a certain behavior. As a consequence, red color is expected to initiate risk-averse behaviors. However, color effects are largely dependent on the prevalent situational characteristics [9]. As mentioned above, for example, in mating situations red can have the opposite effect and activate approach behavior: red increases men’s solicitations of women sitting at a bar [6] and increased courtship behavior in response to personal ads [23]. Some studies also suggest similar effects for online behaviors. For example, banner ads predominantly colored red received more clicks than respective ads in blue [26]. This line of research therefore suggests that red color increases the valence of desirable outcomes and, thus, results in a stronger focus on potential gains.

Taken together, previous findings support two alternative routes through which red might yield its behavioral effects: Red color can elicit either approach or avoidance motivation depending on the current psychological context [32, 33]. In romantic contexts (e.g., dating situations) red tends to induce an approach focus [6, 23, 34, 35], whereas in achievement settings avoidance motivation is typically seen as the primary mechanism triggered by red [4, 5, 11, 28]. The present study focuses on financial risk-taking in an online environment; that is, participants had to decide between two choice alternatives that carried different chances of success. In this context, red color is expected to guide behaviors by focusing the attention on potential losses of a decision [28]. Therefore, we would expect to observe less risk-taking when respondents are faced with red in an online environment. This hypothesis was examined in two web-based experiments that both manipulated task-irrelevant color cues (i.e. the colors were used for aesthetical and decorative reasons but not to convey an explicit meaning) of the online environment.

### Overview of Studies

Color designs of online environments including red cues were hypothesized to affect the willingness to take risks. We conducted two studies that made use of key experimental paradigms in the field of risk-taking behavior. The first study analyzed the effect of red in classic risky choice tasks [36]. The second study highlighted the behavioral consequences of red color in a competitive single-player game with risky outcomes, the Balloon Analogue Risk Task [37], which has been used in a large number of studies in recent years.

## Study I: Risky Choice

The study examined the effect of subtle red color cues on risky choices. Participants were presented with a series of choice dilemmas requiring a decision between two alternatives that differed in their inherent risk (i.e., one option was riskier than the other). It was expected that red color would act as a warning signal of potential losses, resulting in less risky choices. Moreover, this effect was expected to be more pronounced when both choice alternatives were risky as compared to choices including an option with a certain gain because people have a clear preference for outcomes with 100% certainty compared to outcomes which are merely probable [32]. This *certainty effect* should lead to high preferences for the certain option among all participants. Choices involving two risky options, however, are accompanied by much more ambiguity, which could then be influenced by subtle red color cues included in the web design.

### Method

#### Ethics statement

All research reported in this and the subsequent study was approved by the research ethics committee of the Knowledge Media Research Center in Tübingen (approval: LEK 2015/003). The raw data for both studies is given in the supplement S1 File.

#### Participants

The sample consisted of 383 German students (277 women) of different majors (including cognitive sciences, social sciences, and economics) who were invited by email to complete a web-based questionnaire. Their mean age was 23.95 years (*SD* = 5.2). A total of 16 participants failed the test for proper color vision (see below) and were thus excluded from the analyses. All participants who finished the questionnaire were eligible to enter a lottery for four gift vouchers of EUR 20 each.

#### Measures

Eight choice dilemmas were selected from the classical studies by Kahneman and Tversky [32]. Each item involved a dilemma including two choice alternatives that differed in their inherent risk. The risky option was always associated with a greater gain (e.g., more money) than the low risk option. For example, one item (problem 4 from [32]) required a choice between two gambles: Gamble A offered the chance of winning EUR 4,000 with a probability of *p* = .20, whereas gamble B involved a somewhat lower outcome (EUR 3,000) but with a greater chance of winning (*p* = .25). The eight items differed in the objective risk of their outcomes, whereas the expected value was always identical. Four dilemmas (problems 1, 3 5, and 11 from [32]) included low risk options with a certainty of success, *p* = 1.00 (e.g., problem 3 offered either EUR 4,000 with *p* = .80 or EUR 3,000 with *p* = 1.00), whereas the remaining four items (problems 2, 4, 6, and 13) included low risk options with an uncertain probability of success, *p* < 1.00. Participants were instructed to select the more preferable alternative for each dilemma. The number of risky choices for the four dilemmas including a certainty option (*M* = 1.05, *SD* = 1.02) and the four items without a certainty option (*M* = 2.13, *SD* = 1.06) represented the focal variables.

Ishihara’s color test [38] was used to identify deficiency in color vision. The test was comprised of a circle containing dots of different colors and sizes. For individuals with normal color perception a pattern of dots is perceived as showing a number. Individuals who were unable to identify the correct number indicated color vision deficiency and were therefore excluded from the data analyses.

#### Procedure and experimental manipulation

The entire experiment was presented online and was accessed by the participants via the web browser of their home computers. The experiment was self-paced without time constraints. After initial instructions, each of the eight choice dilemmas was presented in random order on a single page. The experiment followed a 2 (within-subjects) x 2 (between-subjects) factorial design. The within-subjects factor distinguished two types of low risk options, either with or without a certainty of success; the between-subjects factor was formed by the color manipulation: at the top of each page the questionnaire included the headline “Online Study” (431 x 120 pixel in size) which was colored either red or gray. The choice of our experimental color manipulation was aimed at increasing the ecological validity of the experiment because the headline of a questionnaire is a likely implementation of incidental color variations (i.e. for merely aesthetic reasons) in applied settings. Moreover, the manipulation did not compromise the usability of the text (e.g., by reducing the readability of the items) but was limited to a task-irrelevant element of the questionnaire. In terms of the hue-saturation-luminosity (HSL) scheme used to represent colors, a “pure” red color (HSL: 0/100/50) and a “pure” gray color (HSL: 0/0/50) were specified. Both colors were matched on luminosity. By random assignment, 178 students were allocated to the condition with the red color and 189 students were assigned to the control condition including the gray logo. Except for the color of the headline the questionnaires were identical and uncolored.

### Results

**Fig 1:**
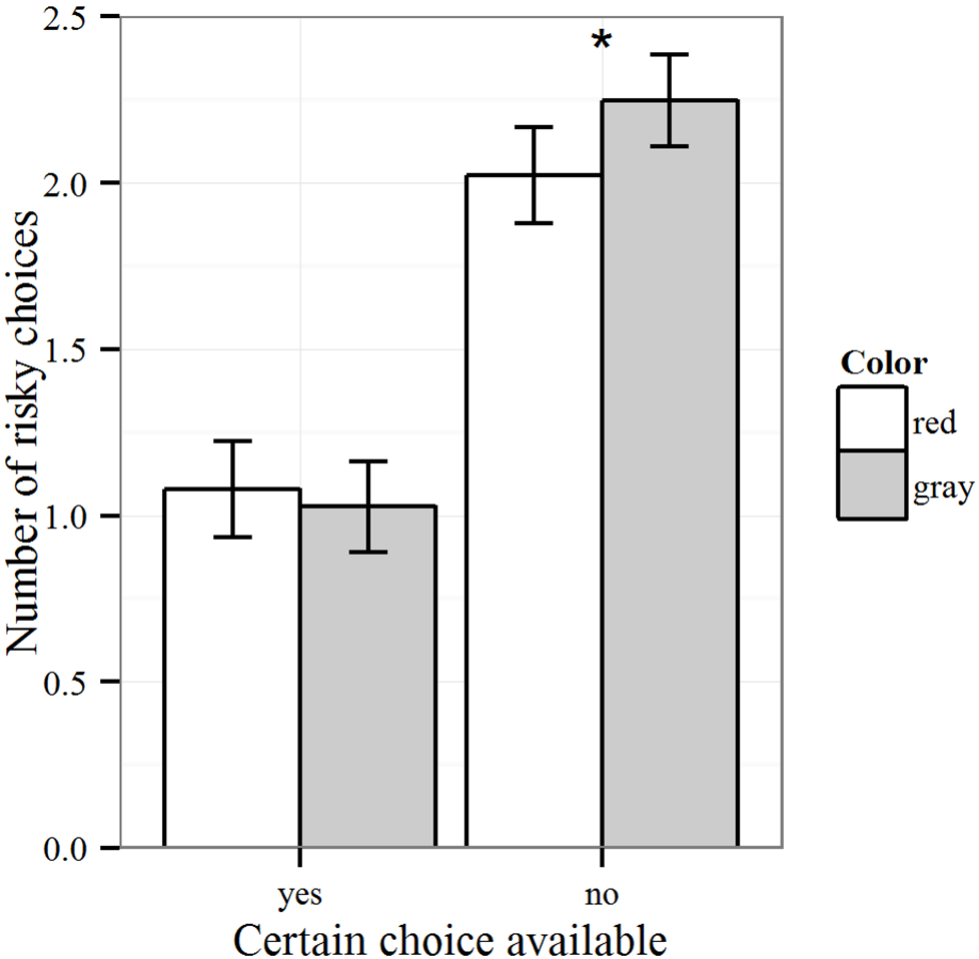
Number of risky choices by color manipulation and risk option (with 95% confidence intervals).

It was hypothesized that the color red would influence risk-taking behavior and thus result in less risky choices than the gray condition. Moreover, this difference was expected to be stronger for risky choices without a certainty option than for choices including an option with a certainty of success. In line with these hypotheses, a 2 x 2 analysis of variance (ANOVA) identified a main effect for the availability of a certain choice alternative, *F*(1, 365) = 233.49, *p* < .001, η_g_^2^ = .22, but no main effect for the color manipulation, *F*(1, 365) = 1.13, *p* = .29, η_g_^2^ = .002. These main effects were qualified by a trend-significant interaction, *F*(1, 365) = 3.82, *p* = .051, η_g_^2^ = .004 (see Fig 1). Analyses of simple main effects revealed homogenous variances, *F*(1, 365) = 0.84, *p* = .36, but a significant difference in the number of risky choices for the color manipulation when no certain choice alternative was presented, *t*(365) = 2.05, *p* = .04, *d* = 0.21 (robustness analyses using a non-parametric test [39] replicated this result with *W* = 18696, *p* = .05). Students chose fewer risky options in the red (*M* = 2.02, *SD* = 1.09) than in the gray condition (*M* = 2.25, *SD* = 1.02). When an option with a certain gain was available, the assumption of variance homogeneity was also supported, *F*(1, 365) = 0.26, *p* = .61, but there was no difference in risky choices between the red (*M* = 1.08, *SD* = 1.02) and the gray condition (*M* = 1.03, *SD* = 1.02), *t*(365) = -0.49, *p* = .62, *d* = -0.05. The color red thus reduced the willingness to take risks in ambiguous situations when all of the choice alternatives posed some risk. In contrast, when a certain choice alternative was available, the color red had no effect.

To examine the potential differences between men and women, we conducted a 2 (certainty option) x 2 (color) x 2 (sex) ANOVA. These analyses did not identify either a significant two-way interaction between sex and the color manipulation, *F*(1, 363) = 0.99, *p* = .32, η_g_^2^ = .002, or a three-way interaction between sex, color and the availability of a certainty option, *F*(1, 363) = 0.36, *p* = .55, η_g_^2^ = .000.

In conclusion, the experiment showed that a task-irrelevant color manipulation of the online environment affected risk-taking behavior. Participants faced with the color red initiated less risky behaviors as compared to participants seeing gray. Moreover, the color effect was more pronounced for choices between two risky options and could not be observed when one choice alternative granted participants a certainty of success. In line with previous research in achievement contexts [31], these results reinforce the link between red and avoidance motivation which leads to risk-averse behaviors in online environments.

## Study II: Competitive Risk Taking

The second study sought to replicate the effect of red color on risk-taking behavior and aimed to extend the insight obtained from the previous experiment by examining actual risk-taking behavior in a competitive task. Instead of making choices between hypothetical outcomes, participants engaged in a game-like contest that required risk behavior to maximize one’s outcome. In line with the previous results, it was expected that the color red would lead to less willingness to take risks as compared to a control condition.

### Method

#### Participants

A total of 144 students (97 women) of diverse majors were invited to participate in a web experiment. Their ages ranged between 17 and 35 years (*M* = 22.73, *SD* = 3.84). Two participants were identified with a suspected deficiency in color vision and were thus excluded from the analyses. Participants were eligible to earn a gift certificate worth EUR 20.

#### Material

**Fig 2:**
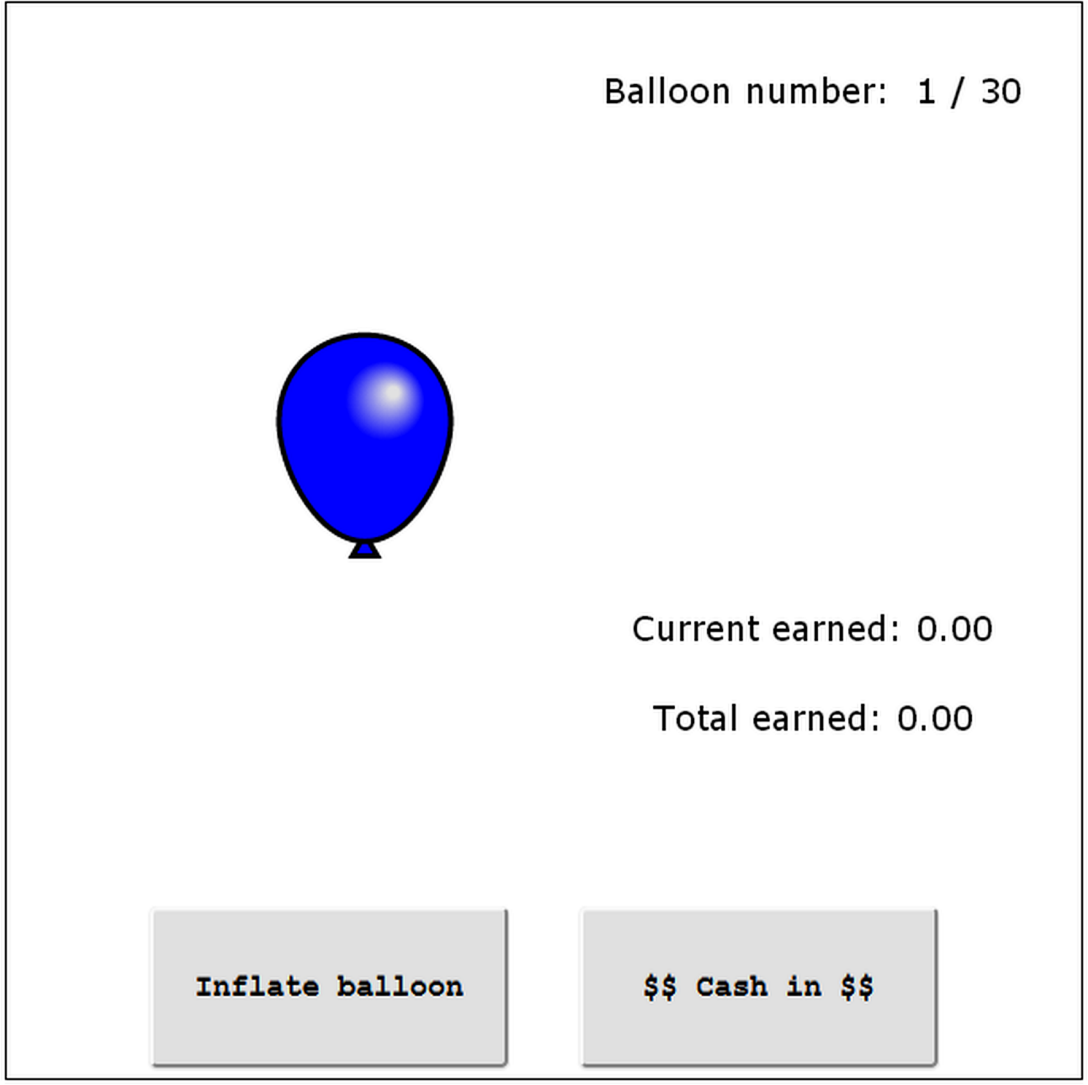
The Balloon Analogue Risk Task.

Risk-taking behavior was measured with the Balloon Analogue Risk Task (BART; [33]), which has been shown to be a valid indicator of real-world willingness to take risks in adolescents [40] and adults [41]. In this task, participants were presented with a series of 30 balloons on the computer screen (see Fig 2) and offered the chance to earn points for inflating the balloons. For each click on a button the current balloon was inflated and the participant received half a point. However, with each click the risk of the balloon bursting also increased. Following the procedure in [33], the probability of the balloon bursting at the first click was 1 / 64. With each subsequent click this probability increased by one increment (e.g., to 1 / 63, 1 / 62 and so on). Thus, each pump was associated with a greater risk but also a greater reward. If a balloon burst, the points acquired for the current balloon were lost. However, instead of continuing to inflate a balloon, participants could choose to collect the points already acquired for the balloon and end the current trial. In order to motivate participants to maximize their points acquired across the 30 trials of the BART, they were informed that the individual achieving the highest total score would receive the gift certificate. For each participant the total number of burst balloons was the focal variable of interest that indicated individual differences in risk taking behavior [33]. Moreover, to examine whether risk-taking also resulted in a competitive advantage we also calculated the total number of points collected across all trials.

#### Experimental manipulation

The entire study including the instruction and the BART was presented via the Internet as an unproctored, self-paced experiment. The experiment manipulated the color of the balloons in the BART. Half of the balloons were red, whereas the other half was blue, yielding a one-factorial within-subjects design. For each participant, the red and blue balloons were presented in random order. Again, we used “pure” colors for red (HSL: 0/100/50) and blue (HSL: 240/100/50). Both colors were matched in terms of saturation and luminosity. Except for their color the balloons were identical; thus, red and blue balloons had the same probability of bursting.

### Results

**Fig 3:**
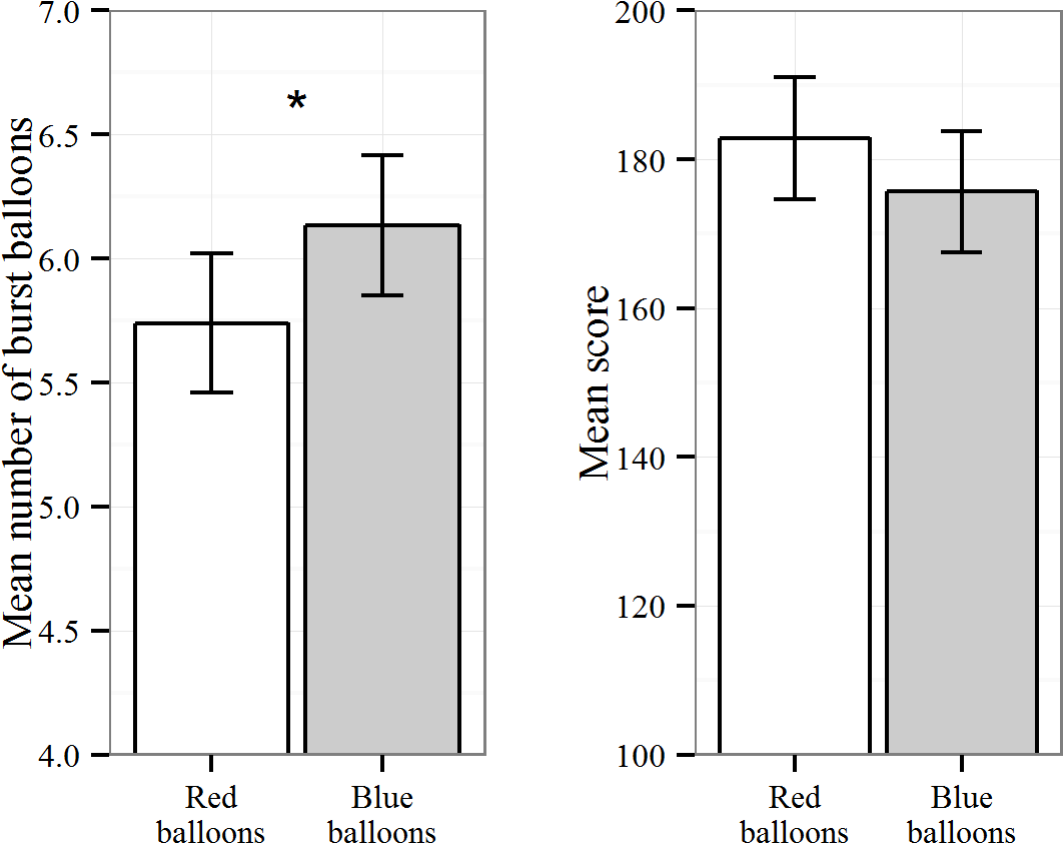
Number of burst balloons and total scores by balloon color (with 95% confidence intervals).

Following the results of the previous study, it was hypothesized that the color red would lead to less willingness to take risks, which would manifest in two indicators of BART performance. We expected participants to over-inflate fewer red balloons than blue balloons. Consequently, fewer red balloons should have burst than blue ones. In line with this hypothesis significantly fewer red balloons burst (*M* = 5.74, *SD* = 2.46) than blue balloons (*M* = 6.13, *SD* = 2.27), *t*(141) = -1.96, *p* = .03 (one-tailed), *d* = -0.16 (robustness analyses using a non-parametric test [39] replicated this result with *V* = 2867, *p* = .04). Sensitivity analyses did not identify differential effects for men and women, *p* > .20. Moreover, the effect of the color on the number of burst balloons could have also translated into a competitive advantage in the BART. Descriptive analyses showed the expected trend (see Fig 3): Participants scored higher on red balloons (*M* = 182.82, *SD* = 58.40) than on blue balloons (*M* = 175.68, *SD* = 58.54). However, this difference failed to reach significance, *t*(141) = 1.22, *p* = .11 (one-tailed), *d* = .12. Although the score differences observed moved in the expected direction, the respective effect was rather small.

Taken together, the second study corroborated the results from the previous experiment. A rather unobtrusive color variation in the design of the balloon task influenced the risk behavior of the respondents. Red color as a frequent signal of danger led people to more cautious pumping behavior, which, in turn, resulted in fewer burst balloons.

## General Discussion

Research on the psychological consequences of color in our surroundings has gained unprecedented momentum in recent years. Particular emphasis has been given to the color red, which can have different implications in different contexts [9, 10, 32, 33]. On the one hand, red elicits avoidance-related affect, cognition, and behavior in the context of competition and test taking. Red activated avoidance motivation in an achievement situation [31], and increased the salience of financial losses as compared to financial gains [28]. On the other hand, red elicited approach-related affect, cognition, and behavior in the context of mating [6, 42]. Likewise, red banner ads received more clicks than similar ads in blue [26].

The aim of this work was to examine the influence of red color cues in online risk-taking behavior based on well-established experimental paradigms. Specifically, it was tested whether red color increased risky behavior as was suggested by different lines of research [23, 26, 28, 31]. Including a red (vs. gray) headline in a web-based survey led users to behave in a more risk-averse way (i.e., to choose less risky options) within a classic dilemma paradigm [32], at least under conditions of uncertainty (Study 1). Likewise, users chose a less risky strategy in an online game when the target stimulus was red rather than blue (Study 2, BART, [33]). Both studies provided comparable evidence that including the color red in online environments can decrease the likelihood of users taking online risks that could result in financial losses. These findings are strengthened by the use of two different control colors, which support the conclusion that the observed differences in online risk taking are a consequence of the color red and not the chosen control conditions. Overall, the results of both experiments add to similar findings in achievement settings [4] and support the view of red as an avoidance trigger. Design features incorporating red cues act as a warning signal for danger [27], resulting in more cautious decisions.

Our results make a contribution to both basic and applied fields of research. Beyond the growing field of color psychology [9], our research has substantial relevance for the interdisciplinary field of decision science, as color is a rather novel situational factor in this field [3]. Moreover, our results are informative for research and practice in web design. Based on our results, creators of websites are advised to consider red color whenever their aim is to impede risky decisions among users (e.g., in online banking) and to avoid color red whenever their aim is to facilitate risky decisions (e.g., on betting and gambling websites).

Despite the contributions of our work, its limitations need to be acknowledged. First, our participants made their choices at home on their private computers or wherever they accessed our web-based studies. This naturalistic setting made it impossible to control the color stimuli as strictly as in the lab. Different monitors might have displayed the same color settings slightly differently and we cannot rule out the possibility that some participants worked with malfunctioning displays. Although the online assessment might have increased error variance, we believe that this imponderability does not invalidate our findings, as the potential display variations worked against our treatment. Rather, more tightly controlled settings in the lab might have elicited even stronger effects. Moreover, these results extend a series of findings on the generalizability of color effects [6, 13, 14, 15, 16] to computerized environments. These results demonstrate that red color also influences individuals’ behaviors outside the laboratory in everyday situations. In the future, it would be desirable to also demonstrate similar effects in field studies that yield real consequences for people to further scrutinize the robustness of color’s consequences in different online but also traditional non-computerized (i.e. offline) situations. Second, our results regarding potential interactions between color cues and other factors that influence decision-making are limited. This study focused on a specific domain of risk-taking, namely financial risk-taking. However, color effects are strongly context-dependent (see [10] for an overview of empirical studies): For example, red undermines performance in intellectual and athletic tasks, but increases sexual attraction in dating situations. Similarly, this study showed that even within a certain psychological context subtle situational differences can make a difference. Study 1 showed that color plays a role regarding risky choices in situations that do not provide a certain choice alternative; in contrast, when a choice with a certainty of success was included, red did not have an effect. Future studies are encouraged to examine other color-by-task characteristic interactions as well as interactions between color cues and person factors. For example, personality traits like the need for cognition or the need for affect [43, 44] are known to shape how message characteristics are processed [45, 46]; whether these traits moderate the effects of color cues in a similar way remains to be determined. Similarly, experience and learned associations can weaken or even reverse potential red effects over time [47]. Third, the studies focused on the behavioral consequences of the color red, but did not examine the underlying processes guiding these behaviors. To obtain a deeper knowledge of the processes underlying the color effects, it would be interesting to examine the implied mediation effect of avoidance motivation on risk-taking behavior, thereby replicating previous findings in the field of cognitive performance [31]. Finally, our results are based on European students; members of a western, educated, industrialized, rich, and democratic society [48]. Despite the fact that the recent studies on the effects of color on psychological functioning showed remarkable consistency across countries [11], not enough is known about culture-based boundary conditions of color effects (see [49] for culture-specific color associations with economic judgements). Although the association between red and concepts of danger seems to be cross-culturally invariant (e.g., similar associations were observed in Chinese and US participants [50, 51]), slightly different color associations across culturally diverse societies might somewhat limit the generalizability of the observed red-risk taking link. Studies aimed at an in-depth analysis of potential cross-cultural differences in the psychological effects of red are therefore highly warranted.

In conclusion, two online experiments concordantly demonstrated that seemingly incidental color cues produced substantial effects on individuals’ decision making. The color red resulted in more conservative choices and less risk taking behavior as compared with two control colors (gray, blue). Although the observed effects might be considered small in size, the findings demonstrate that even rather arbitrary color choices in website designs can make a difference in user behavior.

## Supporting Information

S1 File Raw data for both studies. (ZIP)
